# Optimization of CO_2_ Sorption onto Spent Shale with Diethylenetriamine (DETA) and Ethylenediamine (EDA)

**DOI:** 10.3390/ma15238293

**Published:** 2022-11-22

**Authors:** Asmau Iyabo Balogun, Eswaran Padmanabhan, Firas Ayad Abdulkareem, Haylay Tsegab Gebretsadik, Cecilia Devi Wilfred, Hassan Soleimani, Prasanna Mohan Viswanathan, Boon Siong Wee, Jemilat Yetunde Yusuf

**Affiliations:** 1 Institute of Hydrocarbon Recovery (IHR), Universiti Teknologi PETRONAS (UTP), Seri Iskandar 32610, Perak, Malaysia; 2 Geoscience Department, Universiti Teknologi PETRONAS (UTP), Seri Iskandar 32610, Perak, Malaysia; 3 Department of Fundamental and Applied Sciences, Universiti Teknologi PETRONAS, Seri Iskandar 32610, Perak, Malaysia; 4 Department of Applied Sciences, Faculty of Engineering and Science, Curtin University Malaysia, CDT 250, Miri 98009, Sarawak, Malaysia; 5 Resource Chemistry Program, Faculty of Resource Science and Technology, Universiti Malaysia Sarawak, Kota Samarahan 94300, Sarawak, Malaysia

**Keywords:** amine modification, chemisorption, enhanced sorption, physisorption, spent shale, empirical model

## Abstract

A novel technique was employed to optimize the CO_2_ sorption performance of spent shale at elevated pressure–temperature (PT) conditions. Four samples of spent shale prepared from the pyrolysis of oil shale under an anoxic condition were further modified with diethylenetriamine (DETA) and ethylenediamine (EDA) through the impregnation technique to investigate the variations in their physicochemical characteristics and sorption performance. The textural and structural properties of the DETA- and EDA- modified samples revealed a decrease in the surface area from tens of m^2^/g to a unit of m^2^/g due to the amine group dispersing into the available pores, but the pore sizes drastically increased to macropores and led to the creation of micropores. The N–H and C–N bonds of amine noticed on the modified samples exhibit remarkable affinity for CO_2_ sequestration and are confirmed to be thermally stable at higher temperatures by thermogravimetric (TG) analysis. Furthermore, the maximum sorption capacity of the spent shale increased by about 100% with the DETA modification, and the equilibrium isotherm analyses confirmed the sorption performance to support heterogenous sorption in conjunction with both monolayer and multilayer coverage since they agreed with the Sips, Toth, Langmuir, and Freundlich models. The sorption kinetics confirm that the sorption process is not limited to diffusion, and both physisorption and chemisorption have also occurred. Furthermore, the heat of enthalpy reveals an endothermic reaction observed between the CO_2_ and amine-modified samples as a result of the chemical bond, which will require more energy to break down. This investigation reveals that optimization of spent shale with amine functional groups can enhance its sorption behavior and the amine-modified spent shale can be a promising sorbent for CO_2_ sequestration from impure steams of the natural gas.

## 1. Introduction

Carbon dioxide (CO_2_) is the most common greenhouse gas and is recognized to be one of the driving factors of climate change and global warming. Currently, several countries promote reducing greenhouse gas emissions and safeguarding the global climate. However, anthropogenic CO_2_ emissions are the largest factor, contributing about 63 percent of the total temperature impact from all greenhouse gases [1,2], and emissions of carbon dioxide in the atmosphere peaked at a value of 418.19 ppm in Jan 2022 from 403 ppm in 2016 [3]. CO_2_ emissions are becoming an utmost concern as a result of technological advancement and widespread demand for fossil fuels, which are attributed to global warming and air pollution. In recent decades, the development of fossil fuels from unconventional reservoirs has been widely employed due to the rapid depletion of conventional resources, and most of these unconventional reservoirs, such as shale deposits, coal-bed methane, and ultra-tight wells, have high CO_2_ concentrations [4,5]. During the combustion of these fossil fuels [6] to generate energy, several releases of CO_2_ gas into the atmosphere occur and this emission of CO_2_ from fuel combustion hit about 36.7 billion metric tons per year in 2019 and is estimated to rise to about 38 billion metric tons by 2030 [3]. Hence, there is an imperative need to reduce CO_2_ emissions and also obtain clean energy (energy free from CO_2_ impurities) by applying suitable strategies in developing innovative low-cost capture, sequestration, and several appropriate technologies for extensive gas separation and purification processes. One of the most common technologies widely implemented for this purpose is the “sorption process”, which is defined as the deposition of molecular species in/onto the surface of a sorbent [7].

Several studies were issued to improve the sorption performance of sorbents to act as both physisorbents and chemisorbents [8,9]. This has been made possible by introducing a new functional group to the surface of the sorbents through a chemical modification process known as “functionalization” [10]. The new functional group acts as a support for the sorbent to chemically react with the sorbate, thus enhancing its sorption performance. The widely employed functional groups are the oxygen [11] and nitrogen [12] donors, with the nitrogen donor being reported over the years to be more effective due to its high affinity for CO_2_. The main sources of the nitrogen donor reside in the amine group and include monoethanolamine (MEA), ethylenediamine (EDA), diethylenetriamine (DETA), tetraethylenepentamine (TEPA), triethanolamine (TEA), etc., which have been widely studied and approved due to their environmentally friendly behavior and high sorption rate during industrial usage [13]. According to Wang et al. [8], tetraethylenepentamine (TEPA) was incorporated into a coal residue through the wet impregnation method and found that at 60 °C, the modified coal increased in its quantity of CO_2_ sorbed from 2.68 to 3.39 mmol/g and that the partial pressure of CO_2_ does not affect the sorption capacity of the modified sample. Additionally, Tejavath et al. [14] created amine-modified zeolites for CO_2_ sorption with DETA, EDA, MEA, and TEA. They observed that the DETA-modified zeolites had the highest sorption capacity of 1.69 mmol/g at elevated temperature (75 °C) and further revealed that as amines fill the pores following functionalization, the pore volume, surface area, and other textural characteristics of the sorbent that affect the material’s physisorption capability diminish. As a result, the surface area decreases. Therefore, it is more probable that chemisorption would dominate the sorption process than physisorption after functionalization [15].

However, most existing solid sorbents such as zeolite, silica gel, etc., are relatively expensive, have a short regenerative lifespan, and can easily react or interact with other chemicals [16,17]. Hence, the investigation of sustainable and cost-effective solid sorbents is paramount. Many researchers have studied the sorption behavior of geological formations such as shale [18], coal [19], and coal residues [9] for CO_2_ and they have shown good performance due to their abundant physicochemical properties. In addition, some studies have reported the sorption performance of spent shale (shale residue) for volatile organic compounds such as toluene, benzene [20], and phenolic compound [21] as it reduces production costs since it is widely available as waste. However, no report thus far has investigated its CO_2_ sorption behavior. Therefore, this research aims to investigate the effect of amine modification using diethylenetriamine (DETA) and ethylenediamine (EDA) on the sorption performance of spent shale for CO_2_ capture.

The objectives of this research are in three main parts. Firstly, we analyze the effect of DETA and EDA on the physicochemical characteristics of the spent shale through the use of SAP, FESEM, FTIR, TPD, and TGA analyzers. Further study on CO_2_ sorption is conducted through the use of volumetric sorption techniques. Finally, the experimental data collected are modulated with the two- and three-parameter isotherm and kinetic and thermodynamic models to evaluate the sorption behavior mechanism of the modified spent shale at the operating condition.

## 2. Materials

Four shale samples USQ 06, USM 3, USM 05, and US 6 from the Marcellus Formation, USA, with high percentages of TOC (total organic content) (17.9, 16.2, 13.8, and 12.1, respectively) were pyrolyzed and modified with two amine compounds to investigate their CO_2_ sorption performances. Nitrogen (N_2_) gas was used for pyrolysis, regeneration, and purging of the samples. Diethylenetriamine (DETA) and ethylenediamine (EDA) solutions with over 99.997% purities were utilized for modification based on their abilities previously reported. CO_2_ and helium gases of high purity were obtained for adsorption and desorption measurements, respectively. Further equipment included a water shaking bath, a PROTHERM 5-tube furnace for heating, and a mortar grinder for pulverization of the samples.

## 3. Experimentation

The four Marcellus shale samples USQ 06, USM 3, USM 05, and US 6 were cleaned and dried at 60 °C before being crushed into grain size below 0.15 mm to eliminate any impurity present. An amount of 100 g of each powdered sample was transferred into a ceramic crucible and placed in an automated PROTHERM 5-tube furnace for the pyrolysis process. The N_2_ gas was passed into the tube furnace for 30 min to replace the air and moisture, after which its temperature was increased to 800 °C at the rate of 2 mL min^−1^ for 4 h. The cooled char generated was designated as USQ 06_800C, USM 3_800C, USM 5_800C, and US 6_ 800C. Emulating the procedure employed by Almoneef et al. [22], 20 g of each char was added to 60 mL of DETA and EDA separately in conical flasks and stirred continuously in a water shaking bath for 4 h at 70 °C then filtered and washed with 120 mL of methanol. The slurry was finally dried at 80 °C for complete impregnation of the amine functional group to the surface of the samples.

XRD was employed to identify the crystallographic phases of the samples through the XRD patterns obtained under the Malvern Panalytical equipment coupled with Cu Kα radiation (λ = 1.5405 Å) at 2θ angle = 2–70°. The BET theory, which tries to explain the adsorption process of gas molecules on a solid surface and, therein, serves as the fundamental analytical approach for calculating the specific surface area of materials [23,24], was adopted in this study to determine the pore system of all samples under the SAP equipment (ASAP2020). Their surface morphologies were visualized under the SUPRA 55VP FESEM, and Fourier transform infrared (FT-IR) spectra were acquired using a Perkin Elmer spectrometer (model: Frontier 01) within the range of 4000–400 cm^−1^.

TPD was conducted by subjecting the samples to CO_2_ gas under the temperature-programmed desorption, reduction, and oxidation equipment (TPDRO model 1100). Before the measurements, the samples were heated at room temperature (28 °C) with N_2_ gas at 20 mL min^−1^ and held for 5 min followed by the injection of CO_2_ at a 40 mL min^−1^ ramping rate from 28 °C to 75 °C, which was held for 30 min. This was followed by purging in helium gas at 10 mL min^−1^ from 50 °C to 500 °C, held for 30 min at the peaked temperature to complete the desorption process. In addition, the decomposition kinetics of the oil shale kerogen along with the thermal stability of the samples were determined by the Perkin Elmer spectrometer (model: STA6000).

**Figure 1:**
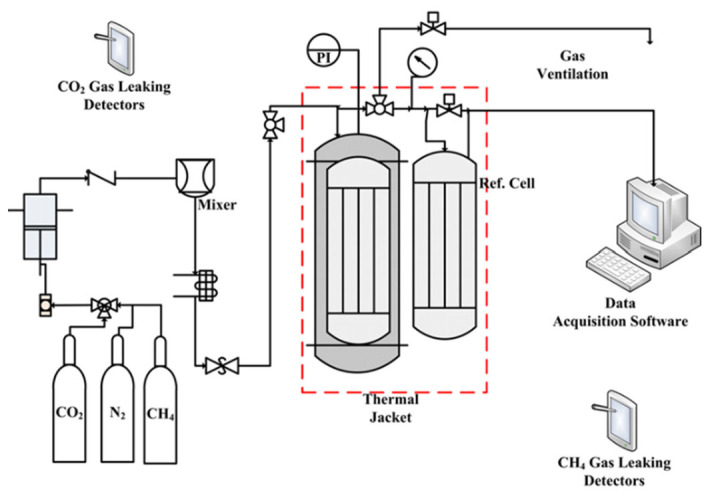
A schematic diagram of the volumetric sorption measurement setup.

Furthermore, CO_2_ gas sorption capacity on all raw, spent, and modified spent shale samples were assessed through a volumetric technique using two reference cells with a flow rate of 600 ± 0.03 mL/min (600 cm^3^/min) and maximum pressure of up to 8 ± 0.05 MPa (78.95 atm). The setup comprised a sample cell, pressure gauge, reference cell, 3-way valve, temperature controller (thermal jacket), and heating oven, as shown in Figure 1. The pressure transferred inside the sample cell was controlled using an electrical pressure transducer (PI). The temperature inside the sample cell was measured using the thermocouples on the sorption system. The experimental setup of the gas sorption system is demonstrated in Figure 1. The CO_2_ sorption measurement began with the degassing process with N_2_ gas for about 30 min to remove any volatile compounds from the surface of the shale samples followed by the injection of CO_2_ onto each sample in the cell. The experiment was conducted in 8 sections from 1 to 8 MPa at an increment of 1 MPa per section and the equilibrium isotherms were determined at three temperatures (30 °C, 50 °C, and 70 °C) to examine the effect of increasing temperature on CO_2_ sorption capacities of the amine-modified samples. Thus, the duration of every single section (pressure) was maintained for 2 h after the equilibrium cell remained constant, indicating that the reaction had reached an equilibrium state.

The amount of CO_2_ gas injected into the sample cell was calculated based on the change in pressure, temperature, and compressibility factor using the Peng–Robinson derivatives in Equation (1):

(1) $$n_{{\mathrm{CO}}_{2}}=\frac{V_{{\mathrm{CO}}_{2}}}{\mathrm{RT}}\left(\frac{P_{i}}{Z_{i}}\right.-\left.\frac{P_{f}}{Z_{f}}\right)$$

where V_CO_2__ is the volume of the CO_2_ gas injected, R is the gas constant, T is the operating temperature, P_i_ and P_f_ are the pressure at the initial and final stages, respectively, and Z_i_ and Z_f_ are the corresponding compressibility factors at the initial and final operating pressures, respectively.

All experimental results obtained from the CO_2_ sorption measurements were correlated and modulated with selective isotherm models that have been widely employed based on their good prediction—namely those of Langmuir, Freundlich, Sips, and Toth—to predict sorption behavior. Furthermore, kinetic models, namely pseudo-first-order, pseudo-second-order, and intraparticle diffusion models, were also employed to determine the mechanism behind the rate of sorption and the thermodynamic parameters (Gibbs free energy, enthalpy, and entropy change) to evaluate their behaviors as the temperature increases. Table 1 presents the equations of all empirical models and the parameters employed.

## 4. Results and Discussion

### 4.1. Mineralogical Content

**Figure 2:**
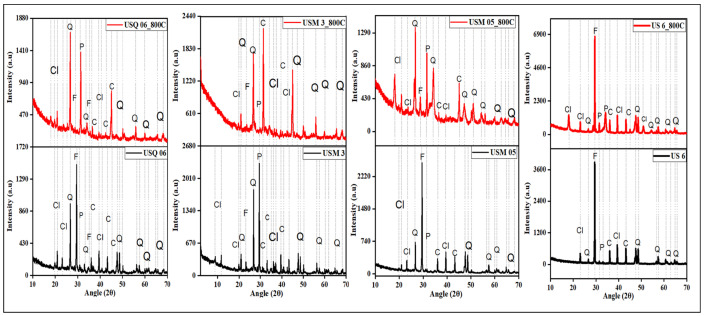
Impact of pyrolysis on the mineralogical content of the shale samples. L–R (USQ 06, USM 3, USM 05, and US 6), Cl = clay, Q = quartz, F = feldspar, P = pyrite, and C = carbonate minerals.

The four shale samples contain clay minerals, feldspar, quartz, pyrite, and carbonate minerals (Figure 2) in both raw and spent shale samples. There are variations in the peak angles of some minerals in the treated samples, especially the clay minerals, which is a result of decomposition in their crystallinities [39]. In addition, the decomposition of some clay minerals at a higher temperature such as kaolinite to metakaolin (high-temperature kaolinite) has been reported to influence their potentiality as commercial materials due to their high reactivity [40,41,42].

However, minerals such as illite, feldspar, and quartz with little or no decomposition due to their high thermal stabilities, together with the cation exchange capacity and inner-sphere complexation, can still maintain their possible vital role in influencing the sorption capacity of shale [43].

### 4.2. Porosity

**Figure 3:**
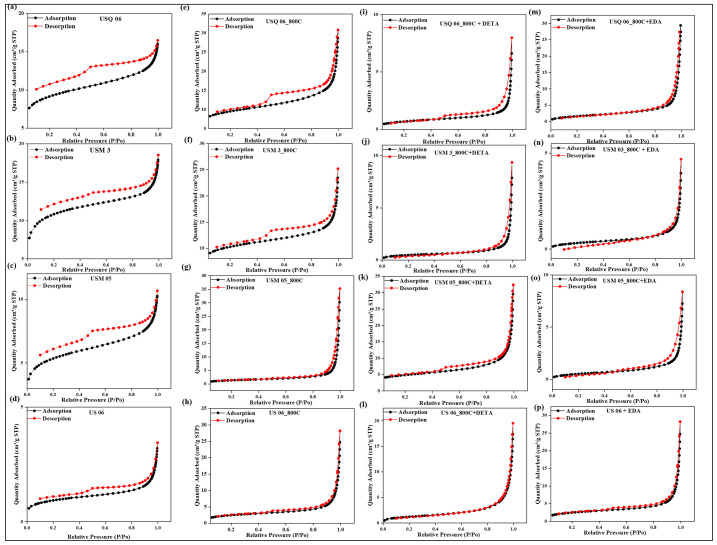
Impact of various treatments on the N_2_ Isotherm curves of Raw shale (**a**–**d**), Spent shale (**e**–**h**), DETA-modified spent shale (**i**–**l**), and EDA-modified spent shale (**m**–**p**).

Classifications of the surface area, pore volume, and pore sizes of the raw, spent, and modified spent shale are presented in Table 2. It was observed that the specific surface area of the spent shale increased drastically after pyrolysis but decreased after amine modification across all samples, as illustrated in Table 2. These changes indicate that the pyrolysis process has created more new pores and enlarged the original pores, thus increasing the surface area. This could be due to the escape of volatiles, water extraction, and mineral decomposition during the pyrolysis process [8,44]. During the amine modification, the nitrogen functional groups have impregnated the surface of the spent shale samples, thus occupying the micro- and mesopores. This led to the reduction in the surface area; however, the macropores’ diameter increased. Similar findings were reported by Chen and Lu [45]. Furthermore, Figure 3 shows the N_2_ adsorption/desorption curves for all samples at relative pressure and temperature of nitrogen. It was observed that they are quite similar. The curves have an inverted “S” form, despite some minor variances. According to the IUPAC and BET isotherm categorization system, these curves are of type II with an H_2_ hysteresis loop, showing microporous and mesoporous structures’ predominance.

It was observed in the raw samples that the branches of the adsorption curves gradually increased until relative pressure (P/P_0_) = 0.8, after which the curves rise steeply but cannot attain an equilibrium up to P/P_0_ > 1.0. This indicates that the molecule of nitrogen contained in the pore condenses below its saturation pressure, a process known as “capillary condensation” [46]. This behavior shows that mesopores are present in the samples. However, the hysteresis loop of the spent shale tends to close up at P/P0 = 0.4 compared to the raw sample, confirming that new pores have been created closer than the previous pores before pyrolysis, which enables desorption of the N_2_ molecules near the adsorption curves and also the increment in the amount of N_2_ sorbed. Similarly, the adsorption/desorption curves of the modified samples are well-connected, revealing that the enlarged pore sizes after modification have aided the pathway of the N_2_ molecules. However, the amount of N_2_ sorbed by the modified samples decreases as a result of the amine group being an N_2_ donor, which cannot adsorb more N_2_ molecules since they are like charges (like charges repel). However, they have been reported to have a high affinity for CO_2_ because it is an unlike charge that they can attract due to electrostatic forces [47].

These characteristics are favorable for CO_2_ sequestration and storage due to the smaller molecular diameter of CO_2_ (0.33 nm) compared to nitrogen (0.36 nm), which enables a larger number of CO_2_ molecules to fit into the increased macropores, mesopores, and the newly created micropores [48].

### 4.3. Morphology

**Figure 4:**
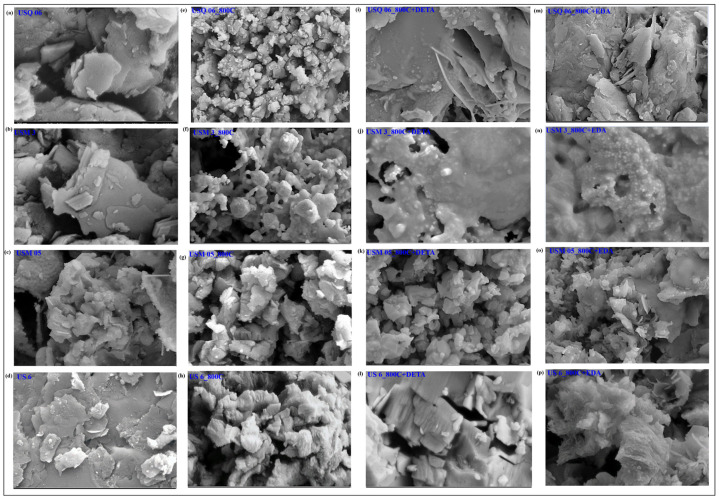
Changes in microfabric of shale after treatments. Raw shale (**a**–**d**), Spent shale (**e**–**h**), DETA-modified spent shale (**i**–**l**), and EDA-modified spent shale (**m**–**p**) at 200 nm resolution and 30 kx magnification.

The raw samples in Figure 4a–d have pores of various sizes coexisting with relatively homogenous areas of lower porosity. In line with the pore structure findings from BET, pyrolysis produced a connection of pores in the spent shale samples that enhances the porosity of the materials, as observed in Figure 4e–h. The pores are responsible for the adsorption of a large volume of N_2_ gas which results in a high surface area. Similarly, the spent shale samples show the existence of inter- and intragranular pores with sharp-edged structures that are maintained in mineral particles such as quartz and feldspar, as well as some intercrystalline pore space connected to the space formed in the clay minerals owing to dehydroxylation [43]. In addition, the modified samples show plane-tunneling pores with broad shapes in Figure 4i–l for DETA-modified samples and in Figure 4m–p for EDA-modified samples.

The amine functional group is assumed to have filled the porous surface and enlarged the average pores present, hence appearing as an occlusion on the surface of the samples. This confirms the results of the BET in Table 2 indicating that the pores have been filled up, leading to a reduction in the surface area. Hence, the photomicrograph revealed that the spent shale and modified spent shale possess higher adsorption sites for CO_2_.

### 4.4. Fourier Transform Infrared Spectroscopy (FT-IR)

**Figure 5:**
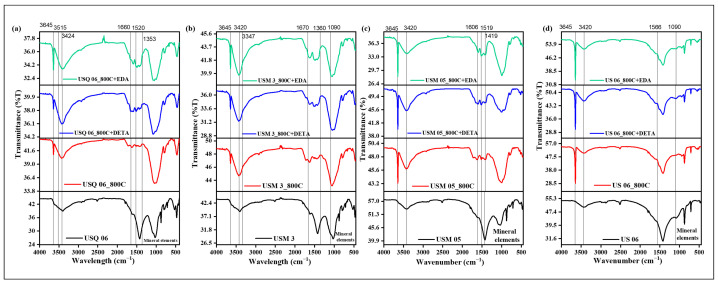
Appearance of new functional groups after the pyrolysis and amine treatments of samples (**a**) USQ 06, (**b**) USM 03, (**c**) USM 5, and (**d**) US 6.

A sharp and narrow peak was observed on all spent shale spectra (Figure 5) at 3645 cm^−1^, corresponding to the hydroxyl group (O–H) stretching on the surface of the samples. This hydroxyl group is associated with Ca(OH)_2_, which is usually formed when a calcium-containing sample is subjected to heat [49,50]. The broad and strong O–H stretching peaks at 3424 cm^−1^ (Figure 5a) and 3420 cm^−1^ (Figure 5b–d), which appeared on all samples, are associated with the sample’s surface affinity for atmospheric water molecules [51]. It was further observed that the modified samples display certain peaks that are typical of the amine group’s C–N and N–H bonds. A weak N–H stretching bond can be seen in the modified samples at 3515 cm^−1^ and 3347 cm^−1^ on samples USQ 06 and USM 3, respectively, near the O–H stretching peak [51,52]. Similar weak and broad peaks at 1660 cm^−1^, 1670 cm^−1^, 1606 cm^−1^, and 1566 cm^−1^ on samples USQ 06, USM 3, USM 95, and US 6, respectively, show the presence of N–H bending with an N–H wag at 1520 cm^−1^ on sample USQ 06 [51]. An asymmetric stretching at 1353 cm^−1^, 1360–1090 cm^−1^, 1519–1419 cm^−1^, and 1090 cm^−1^ correspond to the C–N stretching peak of the amine group [53]. All observed peaks are attributed to the amine functional group used in modifying the samples [53].

### 4.5. Temperature-Programmed Desorption (TPD)

**Figure 6:**
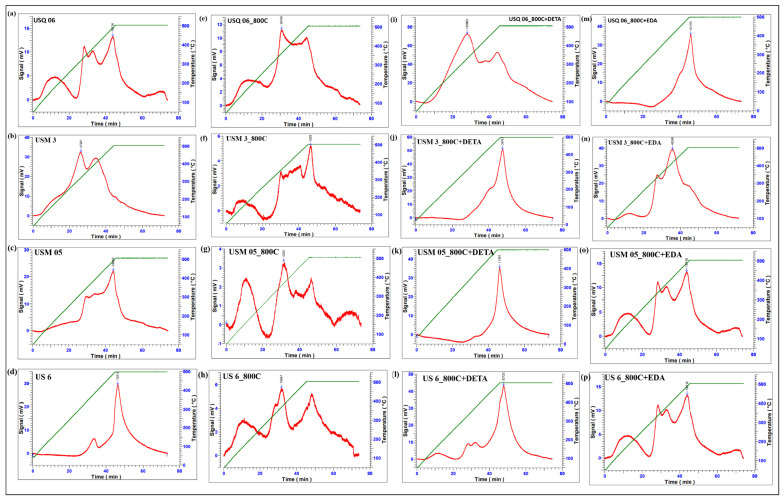
CO_2_ desorption sites at maximum temperature observed on Raw shale (**a**–**d**), spent shale (**e**–**h**), DETA-modified spent shale (**i**–**l**), and EDA-modified spent shale (**m**–**p**).

In the TPD curves shown in Figure 6, it can be observed that there are several adsorption and desorption peaks on each sample, which confirms the presence of active sites for CO_2_ sequestration. All samples showed a strong desorption peak around 450 °C to 500 °C, which corresponds to the maximum temperature (T_max_) at which the chemically sorbed CO_2_ has desorbed [48]. The DETA-modified samples exhibit the strongest desorption peaks with the highest signal strength of ten thousand mV and the highest amount of CO_2_ sorbed, as illustrated in Table 3. This confirms the optimum interaction of the amine group with CO_2_, which will require a large amount of energy to desorb.

### 4.6. Thermal Stability

**Figure 7:**
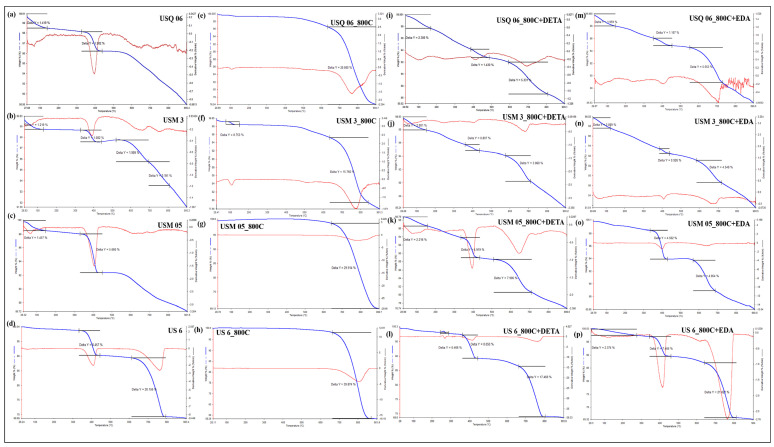
The thermal stabilities of Raw shale (**a**–**d**), spent shale (**e**–**h**), DETA-modified spent shale (**i**–**l**), and EDA-modified spent shale (**m**–**p**) at up to 900 °C.

The raw samples (Figure 7a–d) show weight loss from about 1.2 to 1.4% under conditions of room temperature to about 150 °C, which is attributed to the loss of moisture on the surface or dehydroxylation of clay minerals present in the samples [50]. Another endothermic peak is evident at about 350 °C to 450 °C and is attributed to the breakdown of hydrocarbons in the samples. In the spent shale samples (Figure 7e–h), a significant weight loss of 20.5 to 30% at 650 °C is evidenced by the DTG curve peak at 780 °C. This weight loss is related to the decomposition of the carbonate minerals in the samples. There have been suggestions that the endothermic peaks of carbonate decomposition occur at around 600 °C to 950 °C [54]. Several peaks in moisture, decompositions of amine, and carbonates at temperatures up to 150 °C, 400 °C, and 650 °C, respectively, are present on the DETA- and EDA-modified samples (Figure 7i–p) [12]. After the thermal process, over 80% of the samples remain, which confirms their thermal stability above 900 °C.

### 4.7. CO_2_ Sorption Capacity

**Figure 8:**
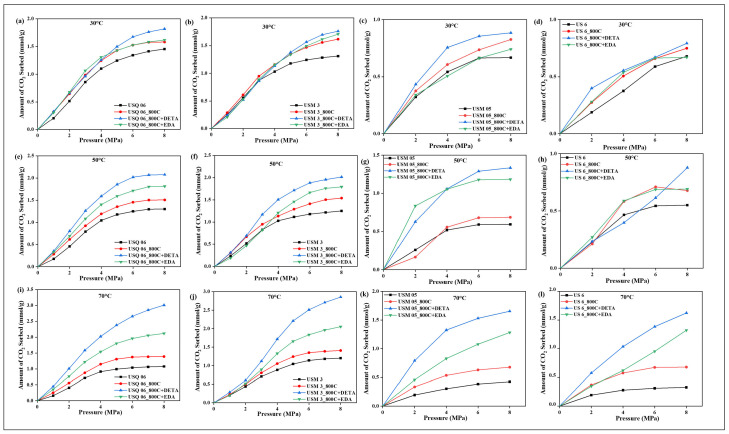
Amount of CO_2_ sorbed relative to the pyrolysis and amine treatments at temperatures 30 °C (**a**–**d**), 50 °C (**e**–**h**), 70 °C (**i**–**l**), and up to 8 MPa pressure.

As shown in Figure 8, as the pressure increased from 1 to 8 MPa, the sorption curves increased across all samples, suggesting that the sorption performance of all samples is similar and increases with an increase in pressure. It was further observed that the sorption performances of all raw and spent shale samples decreased as the temperature increased (Figure 8a–l). However, all modified samples (DETA and EDA) had increased sorption performances with increased temperatures. This implies that the non-modified samples obtained their maximum CO_2_ sorption capacity at the lowest operating temperature, 30 °C, while the modified samples obtained their maximum CO_2_ sorption capacity at the highest operating temperature, 70 °C. The former phenomenon is said to occur when a sorption process is dominated by a physical reaction (physisorption), whereby the molecules of the sorbate (CO_2_) occupy the pores available on the surface of the sorbent with little or no chemical bond involved and tend to diffuse easily when the temperature is increased [55]. The latter occurs when a chemical reaction (chemisorption) is involved, whereby there is a chemical bond between the sorbate and the sorbent, thus requiring a lot of heat to bind together; such reactions tend to be favorable with increased temperature [14].

### 4.8. Effects of Amine Modification on Sorption Performance

**Figure 9:**
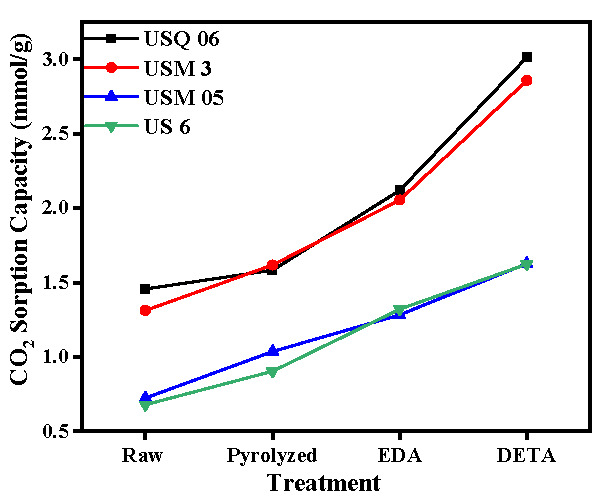
Changes in the CO_2_ sorption capacity of each sample after various treatments.

Further investigation into the sorption results displayed in Figure 5, Figure 6, Figure 7 and Figure 8 showed that the sorption capacity of all shale samples increased after the amine modification process, with the DETA-modified samples having the highest sorption capacity, as displayed in Figure 9.

The shale sample USQ 06 had the highest sorption performance among all four samples. Based on observation, raw sample USQ 06 (Figure 8a) had a sorption capacity of 1.45 mmol/g (mmol of CO_2_/g of the sample), which first increased after the pyrolysis process (as discussed in the previous section) and its spent shale sample, USQ 06_800C, had a sorption capacity of 1.58 mmol/g, but after the amine modification, the sample USQ 06_800C+DETA obtained 3.011 mmol/g and USQ 06_800C+EDA obtained 2.117 mmol/g, which indicated that there was, approximately, a 100% increment in the sorption capacity of the spent shale after being modified with DETA. The EDA-modified samples also showed a higher sorption capacity but not as high as the DETA-modified samples. This is in line with Chen and Lu [45], who investigated the CO_2_ sorption capacity of EDA- and MEA-modified kaolinite and observed that the EDA-modified kaolinite had lower sorption behavior, which could be due to its boiling point (116 °C) being lower compared to MEA and DETA (207 °C in this study). Hence, there is a possibility for the EDA-sample to dry faster with methanol during the drying process. This trend was observed in all other samples: USM 3, USM 05, and US 6 (Figure 7 and Figure 8). However, the high sorption performance of sample USQ 06 can be attributed to its distinguishing characteristics, which have been observed in all analyses conducted, such as high TOC, the porous system under BET, basic strength for CO_2_ under TPD, as well as the high CO_2_ uptake compared to other samples studied under the same operating conditions and analyses. In addition, the possible sorption mechanism of the amine-modified samples and CO_2_, as explained by Wang et al. [8], can be expressed as follows:

(2) $${\mathrm{CO}}_{2}+{\mathrm{RNH}}_{2}↔{\mathrm{RNH}}_{2}^{+}{\mathrm{COO}}^{-}$$

(3) $${\mathrm{RNH}}_{2}^{+}{\mathrm{COO}}^{-}+{\mathrm{RNH}}_{2}↔{\mathrm{RNHCOO}}^{-}+{\mathrm{RNH}}_{3}^{+}$$

where R represents hydrogen or different aliphatic carbon chains. The CO_2_–amine zwitterions are created when the single pair of electrons on the amine RNH_2_ interacts with CO_2_. The zwitterion is deprotonated by another free RNH_2_, which also produces the carbamate salt of carbamic acid and ammonium (Equation (3). These reactions take place within or between amine molecules of the same type or different types of amines [8,56]. The CO_2_ adsorption capacity reported in this work has been compared with related work in the literature. This comparison showed that the amine-modified spent shale in this study has a higher adsorption capacity than some other sorbents that have been reported (Table 4).

### 4.9. CO_2_ Sorption Isotherm Models

**Figure 10:**
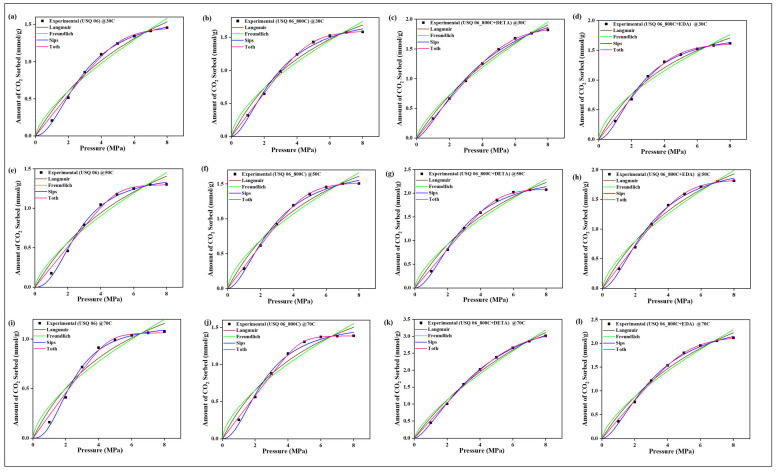
Prediction of the sorption behavior of USQ 06 variants with Isotherm models at temperatures 30 °C (**a**–**d**), 50 °C (**e**–**h**), 70 °C (**i**–**l**), and up to 8 MPa pressure.

**Figure 11:**
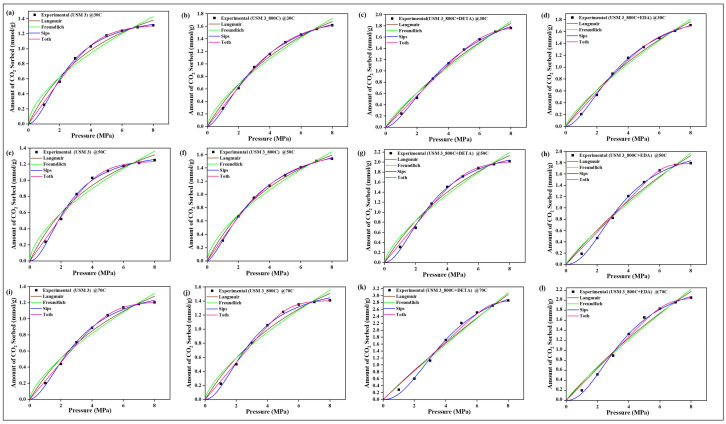
Prediction of the sorption behavior of USM 3 variants with Isotherm models at temperatures 30 °C (**a**–**d**), 50 °C (**e**–**h**), 70 °C (**i**–**l**), and up to 8 MPa pressure.

**Figure 12:**
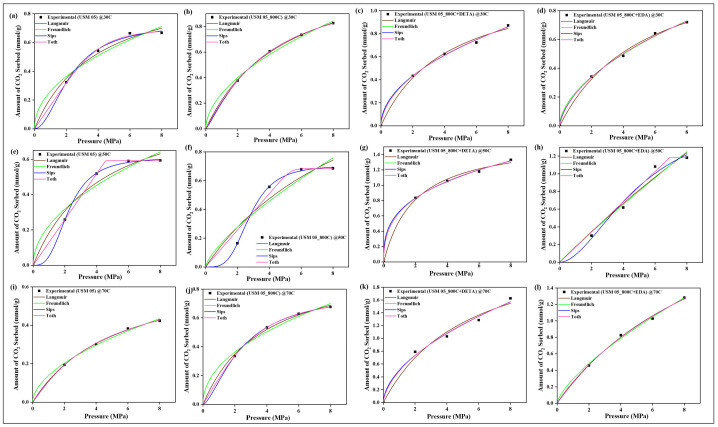
Prediction of the sorption behavior of USM 05 variants with Isotherm models at temperatures 30 °C (**a**–**d**), 50 °C (**e**–**h**), 70 °C (**i**–**l**), and up to 8 MPa pressure.

**Figure 13:**
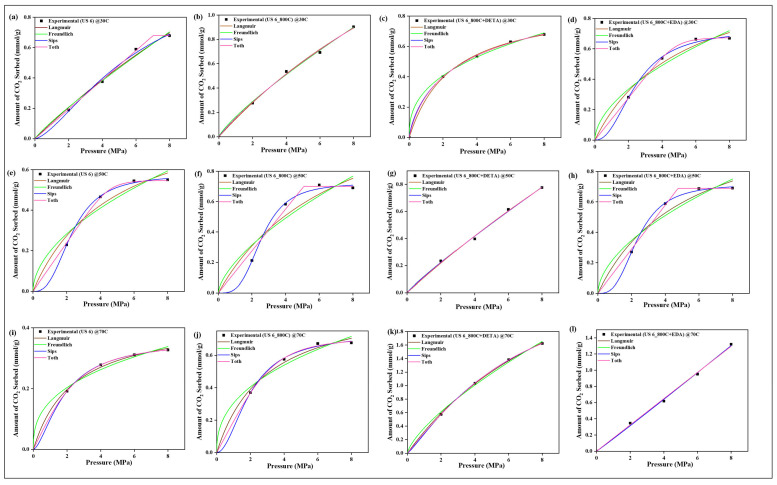
Prediction of the sorption behavior of US 6 variants with Isotherm models at temperatures 30 °C (**a**–**d**), 50 °C (**e**–**h**), 70 °C (**i**–**l**), and up to 8 MPa pressure.

The two-parameter isotherm models of Langmuir and Freundlich and the three-parameter models of Sips and Toth were employed in validating the sorption equilibrium behavior of CO_2_ on the raw, spent, and modified spent shale samples. The experimental data obtained for each sample at the operating temperatures of 30 °C, 50 °C, and 70 °C were all fitted to the four models and their non-linear plots are shown in Figure 10, Figure 11, Figure 12 and Figure 13 for samples USQ 06, USM 3, USM 05, and US 6, respectively. Each isotherm model’s parameters have values obtained from the plots and correlation coefficient constant “R^2^” displayed in Table 5. It was observed that the Langmuir model gave good compliance with the CO_2_ sorption experimental data of all shale samples with R^2^ values ranging from 0.96 to 0.99 (DETA-modified samples having 0.99). This assumes that the CO_2_ molecules have occupied the surface of the shale samples steadily until the entire surface has a monolayer coverage [60]. Furthermore, the values of equilibrium constant k_L_ increased as the temperature increased, which indicated an increase in the binding forces of the CO_2_ molecules on the shale surface as there is acceleration in the mobility of the molecules when the temperature is increased. The values of Langmuir’s maximum sorption capacity (q_m_) of the DETA- and EDA-modified samples increase with elevated temperature, which implies that the accelerated molecules attach to the corresponding amine group due to the attractive bond but easily disperse away on the non-modified samples as there is no amine group leading to the lower values of q_m_ [60].

The Freundlich model also has a good fit with the experimental data but the R^2^ values range from 0.93 to 0.99. Compared to the Langmuir model, the modified samples have R^2^ values below 0.99 but greater than 0.9, which means that they also support a multilayer coverage of sorbate pore filings that further act as an active site for more sorbates [61], while the Sips and Toth models have the best fit with experimental data (R^2^ ranging from 0.98 to 1). The dimensionless heterogeneity factor (n-values) for both models were opposite, which confirms that they agree with the same phenomena. The n-values of the Sips model are greater than unity, while those of the Toth model are less than unity, which implies that they support heterogeneous coverage [61,62]. Hence, based on the isotherm models employed, all samples support monolayer, multilayer, and heterogeneous types of sorption, which indicates that they can actively sequestrate CO_2_ at elevated PT conditions when chemically modified with amine functional groups.

### 4.10. Sorption Kinetics

**Figure 14:**
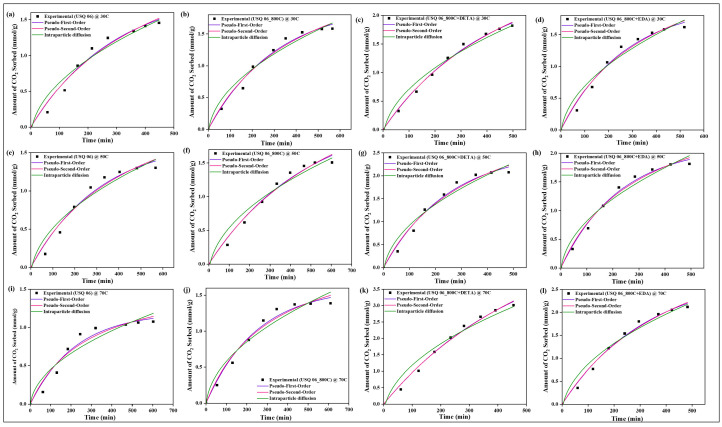
The prediction of sorption rate mechanism on USQ 06 variants at 30 °C (**a**–**d**), 50 °C (**e**–**h**), and 70 °C (**i**–**l**).

**Figure 15:**
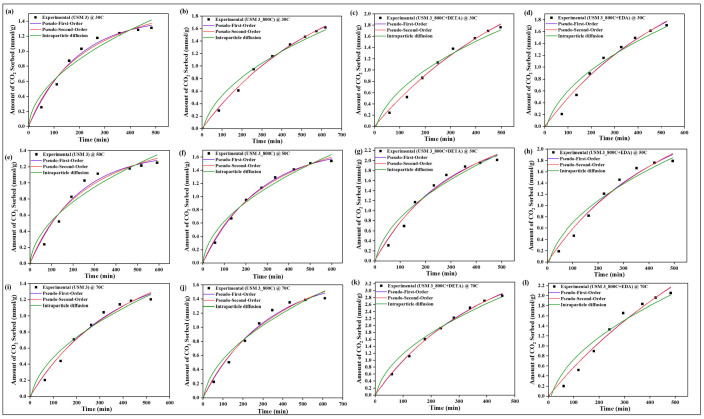
The prediction of sorption rate mechanism on USM 3 variants at 30 °C (**a**–**d**), 50 °C (**e**–**h**), and 70 °C (**i**–**l**).

**Figure 16:**
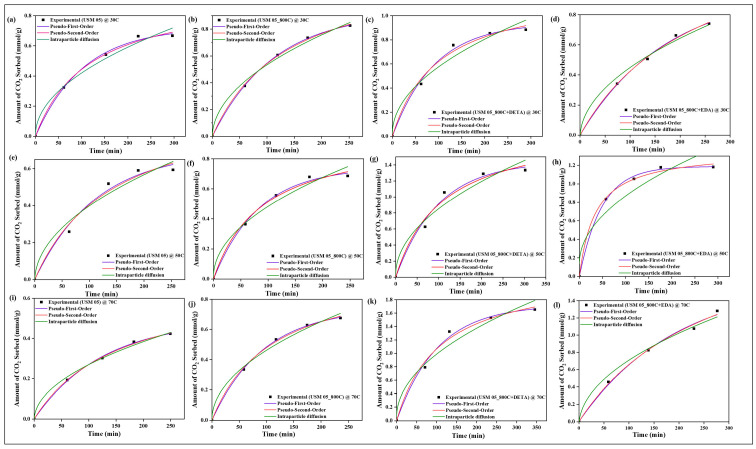
The prediction of sorption rate mechanism on USM 05 variants at 30 °C (**a**–**d**), 50 °C (**e**–**h**), and 70 °C (**i**–**l**).

**Figure 17:**
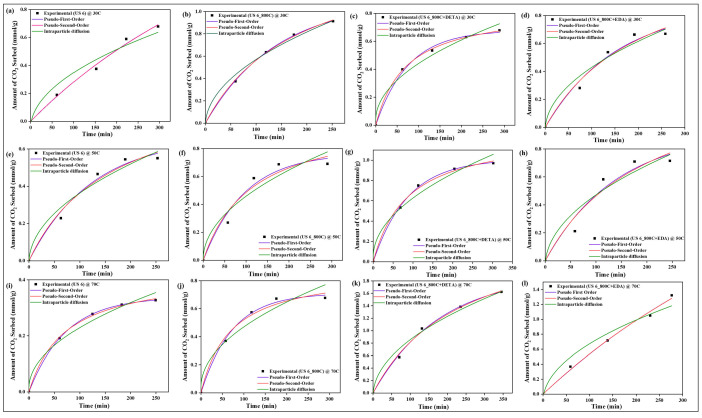
The prediction of sorption rate mechanism on US 6 variants at 30 °C (**a**–**d**), 50 °C (**e**–**h**), 70 °C (**i**–**l**), and up to 8 MPa pressure.

All experimental data were evaluated with three kinetic models, namely pseudo-first-order, pseudo-second-order, and intraparticle diffusion models, to determine the rate of sorption between CO_2_ and the raw, spent, and modified spent shale at operating temperatures of 30 °C, 50 °C, and 70 °C. The parameters of each model, along with their correlation coefficient constant R^2^, obtained from the correlation plots in Figure 14, Figure 15, Figure 16 and Figure 17 for samples USQ 06, USM 3, USM 05, and US 6, respectively, are illustrated in Table 6.

All data have a high correlation coefficient constant with pseudo-first-order and pseudo-second-order models, which confirms their best curve fit as shown in the plots (R^2^ ranging from 0.96 to 0.999). This indicates that the shale samples are in agreement with both physisorption and chemisorption in a similar performance to their monolayer, multilayer, and heterogeneous behavior confirmed by the isotherm models. Furthermore, it was observed that the intraparticle diffusion model does not fit well with experimental data (R^2^ as low as 0.91), and also that the intercept C does not pass through the origin in all cases, which indicates that the rate of sorption is not limited to the diffusion of CO_2_ through the pores but also seems to be associated with some external bodies of reaction [63]. Therefore, the sorption mechanism of the CO_2_ on the modified spent shale can be attributed to both physisorption and chemisorption and not predominantly particle diffusion or chemical reaction.

### 4.11. Thermodynamic Studies

**Figure 18:**
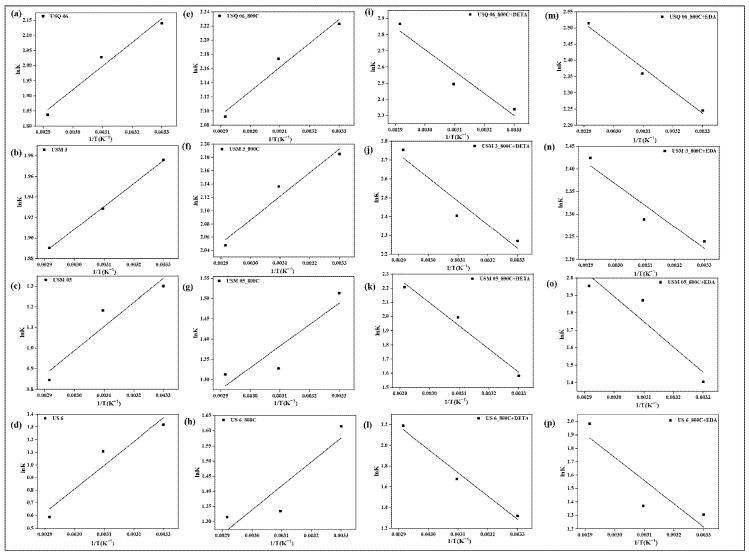
Van ‘t Hoff plots based on the CO_2_ sorbed on Raw shale (**a**–**d**), Spent shale (**e**–**h**), DETA-modified spent shale (**i**–**l**), and EDA-modified spent shale (**m**–**p**).

The insight into thermodynamic parameters such as standard Gibbs free energy (ΔG°), standard enthalpy change (ΔH°), and standard entropy change (ΔS°) are essential to understanding the influence of temperature on the interaction of CO_2_ with sorbents; their related equations are presented in Table 1. Gibbs free energy change is a functional criterion for spontaneity, and determines whether the sorption process is spontaneous or non-spontaneous. If the value of ΔG° is a negative quantity, then sorption has occurred spontaneously at the given temperature. Hence, for significant sorption to occur, the Gibbs free energy (ΔG°) must be negative. As illustrated in Table 7, the ΔG° values obtained at all temperatures are negative for all raw, spent, and modified spent shale samples USQ 06, USM 3, USM 05, and US 6. In addition, the negative values of ΔG° decrease with an increase in temperature for all raw and spent shale samples. This indicates that the sorption process is favorable at a lower temperature. However, the values increase with an increase in temperature for DETA- and EDA-modified samples across all four shale samples, which indicates that the sorption process is favorable at higher temperatures [64]. Hence, amine modification of the samples promotes the sorption of CO_2_ at higher temperatures (reservoir conditions). The ΔH and ΔS were obtained from the slope and intercept of the Van ‘t Hoff plots of ln (k) vs. 1/T, respectively, [38] as shown in Figure 18.

From the values displayed in Table 6, it is observed that the ΔH values were negative for non-modified samples, which confirms an exothermic (physisorption) reaction between the CO_2_ and the sorbent [22], while positive values were obtained for the amine-modified samples, which indicates an endothermic reaction. An endothermic reaction occurs when a large amount of energy is required to desorb an adsorbate (CO_2_) during a sorption process. Invariably, the amine modification has created a chemical bond between the sorbate and sorbents that requires a large amount of energy to break down [65]. It was further observed that the heat of sorption, ΔH, is noticeably higher for the modified samples, which reveals the existence of highly active sites on the surface of sorbents supporting the sequestration of CO_2_. Similarly, negative values of ΔS were obtained for the non-modified samples, which is representative of an associative mechanism of sorption. This implies that there is a decreased disorder at the CO_2_–sorbent interface, causing the CO_2_ molecules to escape from the sorbent surface, thus reducing the amount of CO_2_ sorbed. Further, the amine-modified samples had positive ΔS across all samples, which indicates a dissociative mechanism, i.e., there is an increase in the randomness of the CO_2_ molecules, hence the increased translational entropy at the interface [66].

## 5. Conclusions

Four spent shale samples were modified with DETA and EDA and applied successfully in the sorption of CO_2_ at elevated temperature and pressure. The pore system of the spent shale increased from abundant mesopores to macropores, as the N_2_ group in the DETA and EDA enlarges pores. The evidence of the N–H and C–N bonds associated with the amine functional groups was revealed from the FTIR spectra and enhanced the sorption performance of the spent shale by about 100%. The equilibrium sorption analyses demonstrated that the sorption performance of the modified samples supports monolayer, multilayer, and heterogeneous coverage with the sorption kinetics confirming that the sorption process is not limited to diffusion, and both physisorption and chemisorption also occurred. An endothermic reaction was observed between the CO_2_ and amine-modified samples as a result of the chemical bond, which will require a large amount of energy and heat to break down. These results reveal that optimization of CO_2_ on spent shale with amine functional groups can enhance the sorption behavior of the spent shale, thus validating it as a promising sorbent for CO_2_ sequestration from impure steams of natural gas.

## Figures and Tables

**Table 1:** The equations and parameters of the Empirical models employed in this study.

Models	Equations	Parameters	References
Langmuir	$q_{e}=\frac{q_{m}k_{L}P}{1+k_{L}P}$	Maximum adsorption capacity, q_m,_ (mmol/g)	[25,26]
		Langmuir constant, k_L_, (1/MPa)	
Freundlich	qe=kFP1nF	Adsorption potential, k_F_,	[27,28]
		Adsorption intensity, n_F_	
Sips	qe=qmkSP1nS1+kSP1nS	Maximum adsorption capacity q_m_, (mmol/g)	[29,30]
		Sips constant, k_S_, (1/MPa)	
		Sips exponential, n_S_	
Toth	qe=qmPkT+PnT1nT	Maximum adsorption capacity, q_m_, (mmol/g)	[25,31]
		Toth constant, k_T_, (1/MPa)	
		Toth exponential, n_T_	
Pseudo-first-order	$q_{t}=q_{e}\left(1-e^{-k_{1}\;t}\right)$	Equilibrium uptake, q_e_, (mmol/g)	[32,33]
		Pseudo-first-order constant, k_1_, (min^−1^)	
Pseudo-second-order	$q_{t}=\frac{q_{e}^{2}k_{2}t}{1+q_{e}k_{2}t}$	Equilibrium uptake, q_e_, (mmol/g)	[33,34]
		Pseudo-second-order constant, k_2_, (g/mmol·min^−1^)	
Intraparticle diffusion	$q_{t}=k_{I}\sqrt{t}+C$	Intraparticle diffusion constant, k_I_, (mmol/g·min^−1^)	[35,36]
		Intercept, C	
Thermodynamics	$\Delta G^{{}^{\circ}}=\Delta H^{{}^{\circ}}-T\Delta S^{{}^{\circ}}$	Enthalpy change, ΔH, (kJ/mol)	[37,38]
		Entropy change, ΔS, (kJ/K)	
		Entropy change, ΔS, (kJ/K)	

**Table 2:** Variation in the pore classification after pyrolysis and amine modification processes.

Samples	Surface Area (m^2^g^−1^)	Pore Volume (cm^3^g^−1^)	Average Pore Size (nm)
USQ 06 USQ 06_800C USQ 06_800C+DETA USQ 06_800C+EDA	45.7949 47.9182 8.8351 10.0212	0.0146 0.0328 0.0336 0.0526	5.4257 11.1047 27.6622 20.9626
USM 3 USM 3_800C USM 3_800C+DETA USM 3_800C+EDA	31.2450 33.5156 2.6855 1.9170	0.0144 0.0229 0.0107 0.0052	3.0972 9.3728 35.6702 18.3595
USM 05 USM 05_800C USM 05_800C+DETA USM 05_800C+EDA	16.1194 25.6096 4.8760 3.1368	0.0128 0.0417 0.0144 0.0103	3.5720 18.0721 40.5760 24.0906
US 6 US 6_800C US 6_800C+DETA US 6_800C+EDA	4.7439 6.7906 3.8372 2.4535	0.0048 0.0303 0.0231 0.0029	6.0295 19.1820 24.0804 23.1902

**Table 3:** Variations in the amount of CO_2_ sorbed and its corresponding signal strength with pyrolysis and amine treatments.

Samples	Amount of CO_2_ Adsorbed (μmol/g)	Signal Strength (mV)
USQ 06	40.55448	20,155.49
USQ 06_800C	11.97817	7382.51
USQ 06_800C+DETA	210.65113	123,862.34
USQ 06_800C+EDA	22.75174	18,739.77
USM 3	71.57499	34,761.44
USM 3 _800C	9.44483	5525.20
USM 3 _800C+DETA	79.32970	47,200.97
USM 3 _800C+EDA	64.59448	42,244.61
USM 05	21.1929	11,380.56
USM 05_800C	7.5368	4280.87
USM 05_800C+DETA	43.2398	24,862.82
USM 05_800C+EDA	15.3525	9206.34
US 6	24.9426	16,312.39
US 6_800C	18.6674	10,646.61
US 6_800C+DETA	55.6178	33,722.83
US 6_800C+EDA	31.5258	18,715.74

**Table 4:** Comparison with other amine-modified sorbents for CO_2_.

Sorbents	Amine Group	CO_2_ Sorption Capacity (mmol/g)	Operating Temperature (°C)	References
Spent shale	DETA	3.011	70	This work
Silica MCM 41	DETA	1.43	70	[57]
Zeolite 13X	DETA	1.054	75	[14]
Zeolite Z4A	DETA	0.135	75	[14]
Spent shale	EDA	2.117	70	This work
Kaolinites	EDA	1.495	25	[45]
Silica MCM 41	EDA	1.19	70	[57]
TiO_2_ nanotube	EDA	1.09	30	[58]
UiO-66	PEI	2.41	75	[59]

**Table 5:** The values of isotherm parameters obtained from the curve fit of experimental data at 30 °C, 50 °C, and 70 °C.

Temperature	Isotherm Models	Parameters	USQ 06	USQ 06_800C	USQ 06_800C+ DETA	USQ 06_800C+ EDA	USM 3	USM 3_800C	USM 3_800C+ DETA	USM 3_800C+ EDA	USM 05	USM 05_800C	USM 05_800C+ DETA	USM 05_800C+ EDA	US 6	US 6_800C	US 6_800C+ DETA	US 6_800C+ EDA
30 °C	Langmuir	q_m_ k_L_ R^2^	3.1468 0.1189 0.9809	2.994 0.161 0.984	4.2526 0.1012 0.9918	2.8950 0.1176 0.9797	2.3283 0.1820 0.9840	3.3388 0.1660 0.9924	5.6865 0.0600 0.9911	4.7109 0.0750 0.9892	2.3251 0.0585 0.9947	1.2833 0.2169 0.9989	1.2873 0.2396 0.9949	1.2072 0.0183 0.9967	3.4617 0.0315 0.9928	3.3317 0.0458 0.9976	0.8891 0.3975 0.9994	1.1713 0.1905 0.9831
	Freundlich	k_F_ n_F_ R^2^	0.3720 0.6960 0.9646	0.474 0.625 0.965	0.4398 0.7164 0.9728	0.4996 0.6057 0.9556	0.4092 0.6006 0.9633	0.4233 0.6756 0.9802	0.3469 1.2291 0.9845	0.3572 1.2811 0.9775	0.1451 0.7891 0.9922	0.2825 0.5172 0.9938	0.3087 0.4935 0.9981	0.2323 0.5505 0.9981	0.1124 0.8820 0.9909	0.1640 0.8186 0.9976	0.3128 0.3797 0.9988	0.2284 0.6057 0.9699
	Sips	q_m_ k_S_ n_S_ R^2^	1.6585 0.1224 1.9802 0.9993	1.8823 0.1712 1.7426 0.9968	2.5264 0.1312 1.4686 0.9973	1.8152 0.1781 1.8803 0.9986	1.4872 0.1863 1.8205 0.9988	2.0460 0.1531 1.5566 0.9995	2.4544 0.0926 1.6207 0.9989	2.1537 0.1031 1.7304 0.9998	1.3076 0.0859 1.3001 0.9959	1.0192 0.2304 1.3430 0.9999	3.8935 0.0080 1.9929 0.9981	3.6484 0.0651 1.5502 0.9982	1.1479 0.0658 1.5028 0.9963	5.4818 0.0289 0.9184 0.9977	1.0948 0.3377 0.7622 0.9997	0.7276 0.1282 2.2823 0.9981
	Toth	q_m_ k_T_ n_T_ R^2^	1.4672 0.1887 0.1814 0.9962	1.6056 0.2045 0.1804 0.9999	1.8955 0.1719 0.2072 0.9998	1.6299 0.2161 0.2165 0.9975	1.3316 0.2166 0.2314 0.9985	1.7244 0.1832 0.2883 0.9993	1.8103 0.1553 0.1450 0.9990	1.8086 0.1590 0.2241 0.9965	0.7322 0.1485 0.0857 0.9986	0.9551 0.2212 0.5585 0.9999	2.5651 0.2959 0.1133 0.9980	3.3605 0.0800 0.1922 0.9982	0.6787 0.1425 0.0031 0.9993	15.660 0.0117 0.1929 0.9977	1.1875 0.5347 0.1626 0.9997	0.6738 0.2055 0.1242 0.9998
50 °C	Langmuir	q_m_ k_L_ R^2^	2.7916 0.1268 0.9688	2.9096 0.1548 0.9830	5.2520 0.1375 0.9983	3.6873 0.1372 0.9844	2.2180 0.1925 0.9791	2.8348 0.2203 0.9945	4.4594 0.1142 0.9825	6.8522 0.0889 0.9764	0.9700 0.2322 0.9733	1.7003 0.0969 0.9366	1.6110 0.5076 0.9965	0.8750 0.0206 0.9787	0.9399 0.2048 0.9757	1.4498 0.1352 0.9444	5.6694 0.5199 0.9979	1.4087 0.7411 0.9986
	Freundlich	k_F_ n_F_ R^2^	0.3481 0.6865 0.9684	0.4434 0.6350 0.9644	0.5793 0.6623 0.9577	0.5029 0.6610 0.9680	0.3897 0.6015 0.9667	0.4489 0.6238 0.9812	0.3315 0.8577 0.9687	0.3315 0.8577 0.9687	0.2245 0.5048 0.9564	0.1670 0.7273 0.9205	0.6616 0.3314 0.9994	0.1786 1.0687 0.9768	0.1949 0.5357 0.9599	0.2008 0.6449 0.9255	0.1190 0.9039 0.9982	0.7238 0.2524 0.9950
	Sips	q_m_ k_S_ n_S_ R^2^	1.4371 0.1026 2.3015 0.9979	1.7763 0.1617 1.8012 0.9973	2.4413 0.1419 1.8780 0.9974	2.1656 0.1484 1.7827 0.9976	1.3823 0.1741 1.9547 0.9979	1.9437 0.1892 1.4939 0.9997	2.3584 0.1229 1.9011 0.9985	2.1712 0.0627 2.1510 0.9973	0.6120 0.0918 2.9886 0.9996	0.7087 0.0238 3.6559 0.9995	4.1557 0.0161 2.9373 0.9994	1.7473 0.0517 1.8211 0.9877	0.5750 0.0977 2.7519 0.9994	0.7228 0.0375 3.4513 0.9975	4.7208 0.0025 1.9137 0.9982	1.2901 0.6912 1.3975 0.9990
	Toth	q_m_ k_T_ n_T_ R^2^	1.2991 0.1961 0.1163 0.9943	1.5259 0.2023 0.1700 0.9997	2.1001 0.1951 0.1489 0.9989	1.8420 0.1947 0.1790 0.9992	1.2443 0.2188 0.1924 0.9977	1.7199 0.1981 0.3951 0.9991	2.0341 0.1856 0.1710 0.9974	1.7881 0.1598 0.0656 0.9930	0.5918 0.2189 0.0032 0.9999	0.6845 0.1866 0.0468 0.9740	3.6442 0.2226 0.1027 0.9993	1.1819 0.1445 0.2125 0.9911	0.5471 0.2180 0.1217 0.9808	0.7004 0.1966 0.2017 0.9870	4.0584 0.0030 0.8642 0.9980	1.2766 0.5161 0.6396 0.9990
70 °C	Langmuir	q_m_ k_L_ R^2^	2.0506 0.1624 0.9629	2.6476 0.1640 0.9717	7.7024 0.1847 0.9934	4.5870 0.1786 0.9880	2.5956 0.2213 0.9848	3.1293 0.2661 0.9803	21.5549 0.12045 0.9787	8.7837 0.0912 0.9787	0.7137 0.6861 0.9995	1.0172 0.2604 0.9980	2.6067 0.8830 0.9832	3.0228 0.0902 0.9981	0.4319 0.4179 0.9983	0.9626 0.3412 0.9932	3.9817 0.9871 0.9996	4.0993 1.9553 0.9977
	Freundlich	k_F_ n_F_ R^2^	0.6335 0.3210 0.9371	0.4224 0.6257 0.9486	0.6651 0.7534 0.9851	0.5419 0.6924 0.9745	0.3150 0.6875 0.9698	0.3630 0.6984 0.9945	0.4237 0.9545 0.9760	0.3564 0.8809 0.9725	0.1379 0.5511 0.9967	0.2606 0.4748 0.9915	0.5031 0.8249 0.9927	0.2954 0.4165 0.9975	0.1577 0.3669 0.9922	0.3049 0.4108 0.9835	0.3735 0.3971 0.9973	0.1539 0.0277 0.9979
	Sips	q_m_ k_S_ n_S_ R^2^	1.1501 0.1152 1.4378 0.9974	1.5684 0.1449 1.0682 0.9948	4.0715 0.1166 1.5432 0.9998	2.5937 0.1374 1.7088 0.9988	1.4499 0.1350 1.7917 0.9981	1.6667 0.1220 1.9135 0.9971	3.6019 0.0467 2.1491 0.9972	2.5032 0.0604 2.1114 0.9980	0.6570 0.1954 1.0788 0.9995	0.7898 0.2578 1.5107 0.9999	5.0901 0.0019 1.8175 0.9927	3.5758 0.0784 1.0663 0.9981	0.3587 0.3783 1.5924 1	0.7327 0.2627 1.9436 0.9987	2.9451 0.1069 1.1750 0.9999	2.6598 0.3492 1.0305 0.9979
	Toth	q_m_ k_T_ n_T_ R^2^	1.0642 0.2137 0.1174 0.9931	1.3868 0.2079 0.1019 0.9991	3.2494 0.1604 0.2539 0.9991	2.1644 0.1840 0.1993 0.9992	1.2215 0.1871 0.1685 0.9989	1.4096 0.1868 0.1225 0.9988	2.8616 0.1454 0.0714 0.9986	2.0524 0.1549 0.0921 0.9917	0.6222 0.1922 0.8181 0.9996	0.7510 0.2462 0.4822 1	4.2664 0.0360 0.102 0.9919	4.6906 0.0662 0.7288 0.9982	0.3507 0.3264 0.5039 0.9999	0.7083 0.2739 0.3436 0.9990	2.4255 0.1239 0.5726 0.9999	1.2324 0.0132 0.0155 0.9977

**Table 6:** The sorption kinetics’ parameters obtained from the experimental data at 30 °C, 50 °C, and 70 °C.

Temperature	Kinetic Models	Parameters	USQ 06	USQ 06_800C	USQ 06_800C+ DETA	USQ 06_800C+ EDA	USM 3	USM 3_800C	USM 3_800C+ DETA	USM 3_800C+ EDA	USM 05	USM 05_800C	USM 05_800C+ DETA	USM 05_800C+ EDA	US 6	US 6_800C	US 6_800C+ DETA	US 6_800C+ EDA
30 °C	Pseudo-First-Order	k_1_ q_e_ R^2^	0.0034 1.9211 0.9755	0.0033 1.9653 0.9860	0.0025 2.6050 0.9952	0.0037 1.9793 0.9841	0.0028 1.1726 0.9729	0.0017 2.4869 0.9965	0.0016 3.2340 0.9928	0.0018 2.8463 0.9840	0.0096 0.7225 0.9967	0.0088 0.9297 0.9999	0.0110 0.9412 0.9952	0.0058 0.9625 0.9983	0.0020 1.5237 0.9923	0.0072 1.0955 0.9998	0.0132 0.6784 0.9957	0.0066 0.8639 0.9793
	Pseudo-Second-Order	K_2_ q_e_ R^2^	0.0007 3.0374 0.9720	0.0008 2.9446 0.9824	0.0004 4.1958 0.9939	0.0009 2.9782 0.9792	0.0006 3.4843 0.9819	0.0003 4.1026 0.9957	0.0002 5.6202 0.9921	0.0002 4.8769 0.9828	0.0089 0.9621 0.9948	0.0055 1.3012 0.9989	0.0082 1.2299 0.9895	0.0028 1.4576 0.9980	0.0004 2.6893 0.9922	0.0034 1.6044 0.9989	0.0168 0.8396 0.9993	0.0035 1.3123 0.9753
	Intraparticle diffusion	K_I_ C R^2^	0.0782 −0.1597 0.9408	0.0752 −0.1152 0.9614	0.0903 −0.1931 0.9650	0.0815 −0.1259 0.9527	0.0667 −0.0499 0.9512	0.0706 −0.1774 0.9641	0.0879 −0.2468 0.9473	0.0841 −0.2412 0.9381	0.0412 0.0086 0.9844	0.0539 −0.0053 0.9930	0.0554 0.0225 0.9680	0.0469 −0.0202 0.9893	0.0400 −0.0521 0.9487	0.0593 −0.0219 0.9904	0.0406 0.0352 0.9779	0.0451 −0.0213 0.9583
50 °C	Pseudo-First-Order	k_1_ q_e_ R^2^	0.0032 1.6707 0.9726	0.0021 2.2510 0.9835	0.0040 2.5635 0.9836	0.0044 2.1302 0.9920	0.0044 1.3809 0.9754	0.0038 1.7495 0.9984	0.0035 2.5677 0.9836	0.0024 2.7355 0.9812	0.0089 0.6958 0.9847	0.0121 0.7432 0.9965	0.0101 1.4417 0.9866	0.0205 1.1881 0.9985	0.0081 0.6618 0.9857	0.0103 0.7686 0.9699	0.0115 1.0083 0.9991	0.0071 0.9224 0.9519
	Pseudo-Second-Order	K_2_ q_e_ R^2^	0.0008 2.5822 0.9669	0.0004 3.6919 0.9810	0.0007 3.8523 0.9786	0.0011 3.0945 0.9873	0.0018 1.9386 0.9665	0.0012 2.4936 0.9957	0.0006 3.9648 0.9798	0.0003 4.5572 0.9789	0.0069 0.9933 0.9789	0.0113 0.9777 0.9925	0.0048 1.9034 0.9795	0.0213 1.3595 0.9969	0.0062 0.9655 0.9808	0.0085 1.0343 0.9668	0.0089 1.2783 0.9960	0.0034 1.4222 0.9757
	Intraparticle diffusion	K_I_ C R^2^	0.0651 −0.1381 0.9347	0.0710 −0.1709 0.9464	0.1098 −0.1742 0.9520	0.0932 −0.1154 0.9648	0.0574 0.0484 0.9355	0.0698 0.0731 0.9781	0.1052 0.1987 0.9517	0.0946 0.2534 0.9414	0.0407 −0.0082 0.9634	0.0467 0.0155 0.9763	0.0822 0.0306 0.9568	0.0729 0.1396 0.8987	0.0376 −0.0123 0.9652	0.0452 0.0066 0.9230	0.0583 0.0433 0.9705	0.0508 −0.0367 0.9181
70 °C	Pseudo-First-Order	k_1_ q_e_ R^2^	0.0044 1.1997 0.9601	0.0039 1.6097 0.9840	0.0020 5.1254 0.9919	0.0031 2.8421 0.9903	0.0028 1.6461 0.9860	0.0032 1.7326 0.9879	0.0028 4.0597 0.9987	0.0013 4.5482 0.9792	0.0079 0.4917 0.9990	0.0105 0.7432 0.9996	0.0097 1.7214 0.9938	0.0051 1.6364 0.9942	0.0137 0.3384 1	0.0136 0.7103 0.9957	0.0056 1.9045 0.9975	0.0017 3.4086 0.9941
	Pseudo-Second-Order	K_2_ q_e_ R^2^	0.0021 1.6850 0.9688	0.0013 2.2954 0.9766	0.0014 8.7602 0.9909	0.0004 4.4892 0.9877	0.0007 2.6131 0.9830	0.0009 2.5937 0.9834	0.0003 6.4875 0.9980	0.0001 8.3234 0.9785	0.0089 0.7021 0.9989	0.0088 1.0168 0.9979	0.0042 2.2220 0.9890	0.0015 2.4831 0.9954	0.0324 0.4287 0.9986	0.0163 0.8819 0.9876	0.0015 2.7832 0.9963	0.0002 5.9639 0.9943
	Intraparticle diffusion	K_I_ C R^2^	0.0503 −0.0509 0.9120	0.0655 −0.0754 0.9492	0.1577 −0.3949 0.9482	0.1090 −0.2194 0.9590	0.0611 −0.1293 0.9524	0.0660 −0.1169 0.9588	0.1451 −0.2690 0.97147	0.1076 −0.3512 0.9221	0.0276 −0.0056 0.9950	0.0459 0.0028 0.9907	0.0932 0.0557 0.9647	0.0758 −0.0482 0.9867	0.0216 0.0129 0.9780	0.0429 0.0404 0.9417	0.0902 −0.0484 0.9849	0.0767 −0.0958 0.9518

**Table 7:** Thermodynamic parameters obtained from the experimental data at 30 °C, 50 °C, and 70 °C.

Thermodynamic Parameters	USQ 06	USQ 06_800C	USQ 06_800C+ DETA	USQ 06_800C+ EDA	USM 3	USM 3_800C	USM 3_800C+ DETA	USM 3_800C+ EDA	USM 05	USM 05_800C	USM 05_800C+ DETA	USM 05_800C+ EDA	US 6	US 6_800C	US 6_800C+ DETA	US 6_800C+ EDA
ΔG° (kJ/mol)@ 30 °C ΔG° (kJ/mol)@ 50 °C ΔG° (kJ/mol)@ 70 °C	−123.42 −121.83 −120.24	−127.66 −131.87 −136.09	−131.75 −157.36 −182.97	−128.07 −145.20 −162.33	−113.12 −117.81 −122.50	−125.56 −129.43 −133.30	−127.82 −151.78 −175.74	−127.36 −141.69 −156.02	−76.69 −67.16 −57.63	−85.26 −84.28 −83.29	−92.00 −118.46 −144.92	−83.58 −107.11 −130.64	−78.62 −60.41 −42.20	−90.30 −86.39 −82.48	−73.52 −106.51 −139.50	−69.38 −95.58 −121.78
ΔH° (kJ/mol)	−147.53	−63.83	426.29	327.50	−42.03	−66.94	235.16	89.72	−221.05	−100.23	308.82	272.90	−354.46	−149.48	256.22	131.41
ΔS° (kJ/K)	−0.079	−0.210	1.280	0.856	−0.234	0.1934	1.197	0.7164	−0.476	−0.049	1.322	1.176	−0.910	−0.195	1.649	1.309

## Nomenclature

m^2^/g	Meter squared per gram
ppm	Part per million
mmol/g	Millimole per gram
λ	Wavelength
nm	Nanometer
θ	Theta
mL/min	Milliliter per minute
MPa	MegaPascal
atm	Atmospheric condition
n_CO_2__	Amount of CO_2_
V_CO_2__	Volume of CO_2_
P_i_	Initial Pressure
P_f_	Final Pressure
Z_i_	Initial Compressibility Factor
Z_f_	Final Compressibility Factor
q_e_	Equilibrium uptake
q_m,_	Maximum adsorption capacity
k_L_,	Langmuir constant
k_F_,	Adsorption potential
n_F_	Adsorption intensity
k_S_	Sips constant
n_S_	Sips exponential
k_T_	Toth constant
n_T_	Toth exponential
q_t_	Equilibrium uptake at time t
k_1_	Pseudo-first-order constant
k_2_	Pseudo-second-order constant
k_I_	Intraparticle diffusion constant
C	Intercept
ΔG	Gibbs free energy
ΔH	Enthalpy change
ΔS	Entropy change
P/P_0_	Relative Pressure
